# Eight-Week Vitamin D_3_ Supplementation at 4000 IU/Day Was Not Associated with Further Improvements in Speed or Power Performance in Professional Female Soccer Players: A Randomized Controlled Trial

**DOI:** 10.3390/nu18152460

**Published:** 2026-07-28

**Authors:** Małgorzata Magdalena Michalczyk, Mariola Gepfert, Robert Roczniok, Grzegorz Zydek

**Affiliations:** Institute of Sport Sciences, The Jerzy Kukuczka Academy of Physical Education in Katowice, Mikolowska 72a, 40-065 Katowice, Poland; m.gepfert@awf.katowice.pl (M.G.); r.roczniok@awf.katowice.pl (R.R.); g.zydek@awf.katowice.pl (G.Z.)

**Keywords:** vitamin D, supplementation, female soccer players, strength, speed

## Abstract

**Background:** Vitamin D is involved in musculoskeletal function, but evidence that supplementation improves athletic performance remains inconsistent, particularly in athletes with sufficient baseline vitamin D status. **Methods:** In this double-blind randomized controlled trial, 18 professional female soccer players were randomized during the autumn preparatory period (August–September) to receive vitamin D_3_ (4000 IU/day; *n* = 9) or placebo (*n* = 9) for eight weeks. Outcomes included total serum 25-hydroxyvitamin D [25(OH)D] and 1,25-dihydroxyvitamin D [1,25(OH)_2_D] concentrations, hematological variables, RAST total sprint time, 5- and 30-m sprint performance, and countermovement-jump outcomes. **Results:** At baseline, after summer exposure, 25% of participants had insufficient or deficient 25(OH)D concentrations (≤30 ng/mL). The remaining cohort (75%) had sufficient 25(OH)D concentrations (>30 ng/mL). After eight weeks, no statistically significant between-group differences were observed in vitamin D metabolites, hematological variables, or performance outcomes (Δ25(OH)D: SG +12.4 ± 8.2 ng/mL vs. PG +3.1 ± 6.5 ng/mL; *p* = 0.12). RAST total sprint time (*p* = 0.001) and 30-m sprint performance (*p* = 0.005) improved over time. **Conclusions:** Vitamin D_3_ supplementation at 4000 IU/day for eight weeks was not associated with additional improvements in muscle strength, sprint performance, or countermovement-jump outcomes compared with placebo. Because most participants had sufficient baseline 25(OH)D concentrations, larger trials in female athletes with confirmed vitamin D insufficiency or deficiency are needed to determine whether individualized supplementation or longer intervention periods provide additional physiological or performance-related benefits. The trial was retrospectively registered at ClinicalTrials.gov (NCT07641075) on 8 June 2026.

## 1. Introduction

Vitamin D is involved in several physiological processes relevant to athletes, including skeletal muscle function, calcium homeostasis, immune regulation, and erythropoiesis [1,2,3,4,5,6,7,8,9,10]. These mechanisms provide biological plausibility for a relationship between vitamin D status and exercise performance; however, they do not establish that supplementation directly improves strength, power, recovery, or hematological outcomes. Evidence from randomized controlled trials remains inconsistent, particularly in athletes with sufficient baseline serum 25-hydroxyvitamin D [25(OH)D] concentrations [4,7,11].

Soccer performance depends on repeated high-intensity actions, including sprinting, jumping, kicking, tackling, and rapid changes in direction, interspersed with periods of lower-intensity activity [12,13,14,15,16,17,18]. Although linear speed and muscular power are important determinants of performance, soccer is multidimensional and requires reaction time, perceptual–motor efficiency, agility, and effective decision-making [17,18]. Observational studies have reported associations between lower serum 25(OH)D concentrations and impaired neuromuscular function or physical performance in soccer players; however, these associations cannot be interpreted as evidence of a causal effect of vitamin D supplementation [5,13,14,19,20,21].

Vitamin D has also been implicated in erythropoiesis and immune regulation, which are relevant to oxygen transport, susceptibility to infection, and recovery in athletes. Nevertheless, evidence that vitamin D supplementation produces clinically meaningful changes in hematological indices or post-exercise recovery remains limited and inconsistent [8,9,10]. Therefore, hematological variables should be regarded as exploratory outcomes rather than established indicators of an ergogenic response to vitamin D supplementation.

Randomized trials and recent systematic reviews have reported mixed findings regarding the effects of vitamin D supplementation on strength and power, with potential benefits appearing more likely in athletes with low baseline serum 25(OH)D concentrations [4,7,11,19]. Most studies conducted in soccer players have involved male athletes, whereas evidence concerning professional female players remains scarce. This research gap is relevant because sex-specific physiological characteristics, training demands, nutritional status, hormonal factors, and seasonal variation may influence both vitamin D status and the response to supplementation [2,3,18,22,23].

Female soccer has become increasingly professional, and the physical demands imposed on players have risen accordingly. Nevertheless, female athletes remain underrepresented in sport nutrition and supplementation research [18,22,23]. Randomized controlled trials are therefore needed to determine whether vitamin D supplementation provides physiological or performance-related benefits beyond adaptations associated with regular training, particularly in players who begin an intervention with sufficient serum 25(OH)D concentrations.

Therefore, the aims of the present study were twofold: first, to assess total serum 25(OH)D concentrations in professional female soccer players at the end of the summer period and, second, to evaluate the effects of eight weeks of vitamin D_3_ supplementation at a dose of 4000 IU/day on vitamin D status, sprint and repeated-sprint performance, countermovement-jump outcomes, body composition, and selected hematological variables. We hypothesized that vitamin D_3_ supplementation would increase serum 25(OH)D concentrations and produce greater improvements than placebo in sprint, repeated-sprint, and countermovement-jump performance.

## 2. Material and Methods

### 2.1. Participants

**Figure 1:**
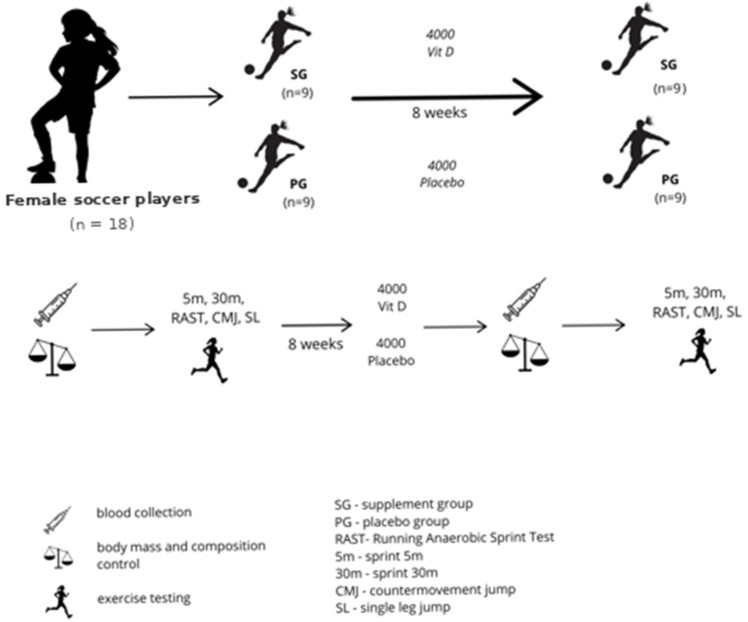
Study design.

The study was conducted between August and September. At baseline (August), 18 professional female soccer players were recruited (height 166 ± 3.4 cm; body mass 58.6 ± 4.2 kg; body fat 17.5 ± 3.0%; muscle mass 28.7 ± 2.5 kg; training experience 7 ± 1 years). All participants represented the same professional club competing in the Women’s Ekstraliga, the highest level of women’s football competition in Poland. The participants included four wide midfielders, four wide defenders, four strikers, four central midfielders, and two central defenders. Participants were randomized in a 1:1 ratio to either the supplementation group (SG, *n* = 9) or the placebo group (PG, *n* = 9) (Figure 1). The randomization sequence was generated using a computer-based random-number generator by an investigator who was not involved in participant recruitment or outcome assessment. Participant enrollment, allocation, follow-up, and analysis are presented in the CONSORT 2010 flow diagram (Figure S1). Allocation was concealed until participants were assigned to their respective intervention groups. The study was conducted in a double-blind manner; neither the participants nor the investigators responsible for performance testing and data analysis were aware of group allocation until completion of the statistical analyses. All participants completed the intervention and were included in the final analyses. The inclusion criteria were female sex, membership in a professional team, regular training, at least six years of soccer training experience, absence of musculoskeletal injuries during the six months before baseline assessment, participation in at least five training sessions per week during the preceding six months, no vitamin D supplementation for at least one month before enrolment, no use of multivitamin preparations containing vitamin D, and absence of gastrointestinal disorders known to impair nutrient absorption. Written informed consent was obtained from all participants before enrolment. The study protocol was approved by the Bioethics Committee (Approval No. 05/2017, 5 December 2017). The trial was retrospectively registered at ClinicalTrials.gov (Identifier: NCT07641075; registered on 8 June 2026).

### 2.2. Study Design and Interventions

This study aimed to optimize vitamin D status by increasing circulating 25(OH)D concentrations and to evaluate whether improved vitamin D availability influences physical performance-related parameters in professional female soccer players. The study was conducted in a country located at approximately 50° N latitude, where seasonal variations in sunlight exposure and reduced UVB availability during certain periods of the year may limit endogenous vitamin D synthesis. The intervention lasted for eight weeks and was conducted during the preparation period for the autumn competitive season (Figure 1). Throughout the study period, the participants followed their regular training program, which included daily soccer training sessions (approximately 2 h/day) and official league matches held on weekends (Saturday/Sunday). Strength and conditioning training sessions were performed twice a week. The detailed training scheme is shown in Table 1. Venous blood samples were collected before and after the 8-week supplementation period for biochemical analyses, including total serum 25(OH)D and 1,25(OH)_2_D concentrations, as well as selected hematological parameters. Body mass and composition were assessed at both time points. Following the standardized warm-up, the performance assessments were conducted in the following order: 5 m and 30 m sprint tests, RAST (Running-based Anaerobic Sprint Test), bilateral CMJa, and unilateral CMJ tests, with 15 min of recovery between successive tests. All sprint tests were performed on an indoor artificial grass surface.

### 2.3. Vitamin D Supplementation Protocol

Participants in the supplementation group received one softgel capsule daily containing 4000 IU (100 µg) of cholecalciferol (vitamin D_3_; NEURO NUTRITION, Poznań, Poland) for eight weeks. The active capsules contained olive oil as a carrier, gelatin and glycerol as components of the capsule shell, and cholecalciferol derived from lanolin. Participants in the placebo group received an identical softgel capsule containing olive oil in the same gelatin–glycerol shell. The supplement and placebo capsules were identical in appearance and were taken once daily in the evening after dinner. A certificate of analysis supplied by the manufacturer accompanied the investigational product and confirmed its declared composition and vitamin D_3_ content. Participants were instructed to refrain from using additional vitamin D or other nutritional supplements during the intervention. The dose of 4000 IU/day was selected on the basis of previous studies in physically active individuals and athletes showing that this dosage can safely increase serum 25(OH)D concentrations while remaining within the established tolerable upper intake level. The eight-week intervention period was selected because previous supplementation studies indicated that this duration is sufficient to induce measurable changes in serum 25(OH)D concentrations. Adherence was supported throughout the intervention by a designated member of the club staff who maintained daily contact with the players, provided reminders, and monitored capsule intake. Participants were instructed to report any potential adverse events or discomfort. No supplementation-related adverse events were reported.

#### Control of Potential Confounding Factors

The participants were instructed to maintain their habitual dietary intake and training routines throughout the study period. No controlled diet was implemented during the study, and individual sun exposure was not monitored. The study was conducted during the late summer/early autumn period in a country located at approximately 50° N latitude, where seasonal variations in sunlight exposure may have affected endogenous vitamin D synthesis. Training schedules were comparable between groups, as all participants followed the same team training program. Individual data on dietary vitamin D and calcium intake, sunlight exposure, skin phototype, sunscreen use, travel history, menstrual cycle phase, hormonal contraceptive use, and menstrual dysfunction were not systematically collected, and this is acknowledged as a limitation of the study.

### 2.4. Serum Vitamin D and Hematological Analyses

Fasting venous blood samples were collected in the morning (approximately 8:00 a.m.) after body mass assessment. A standard automated hematology analyzer was used to determine red blood cell count (RBC), white blood cell count (WBC), hemoglobin concentration (HGB), and the selected leukocyte subtypes. Serum was separated using routine procedures and was either analyzed immediately or stored at −70 °C until analysis. Total serum 25-hydroxyvitamin D [25(OH)D] concentrations were determined using the 25OH Vitamin D Total RIA-CT assay (KIP1971/KIP1974; DIAsource ImmunoAssays S.A., Louvain-la-Neuve, Belgium). The assay measures the combined concentrations of 25(OH)D_2_ and 25(OH)D_3_. The manufacturer-reported intra-assay coefficient of variation was 4.7%, and the inter-assay coefficients of variation ranged from 5.8% to 6.7%. Serum 1,25-dihydroxyvitamin D [1,25(OH)_2_D] concentrations were determined using a competitive radioimmunoassay (1,25(OH)_2_ Vitamin D RIA-CT, catalogue no. KIP1929; DIAsource ImmunoAssays S.A., Louvain-la-Neuve, Belgium), following solvent extraction and cartridge-based separation according to the manufacturer’s instructions. The manufacturer-reported intra-assay coefficients of variation ranged from 6.8% to 7.4%, and the inter-assay coefficients of variation ranged from 11.3% to 12.7%. Assay analytical performance: the manufacturer-reported intra-assay/inter-assay coefficients of variation were 4.7%/5.8–6.7% for total 25(OH)D and 6.8–7.4%/11.3–12.7% for 1,25(OH)_2_D.

### 2.5. Body Mass and Body Composition Evaluation

After an overnight fast, the participants reported to the laboratory in the morning for body mass assessment. Participants were instructed to maintain their habitual hydration status before testing; however, hydration status was not directly standardized or assessed before measurements. Body composition and body fat content were evaluated using multifrequency bioelectrical impedance analysis (MF-BIA) with an InBody 720 device (Biospace Co., Ltd., Seoul, Republic of Korea). Measurements were performed under laboratory conditions according to the manufacturer’s instructions.

### 2.6. The 5-m and 30-m Sprint Tests

**Figure 2:**
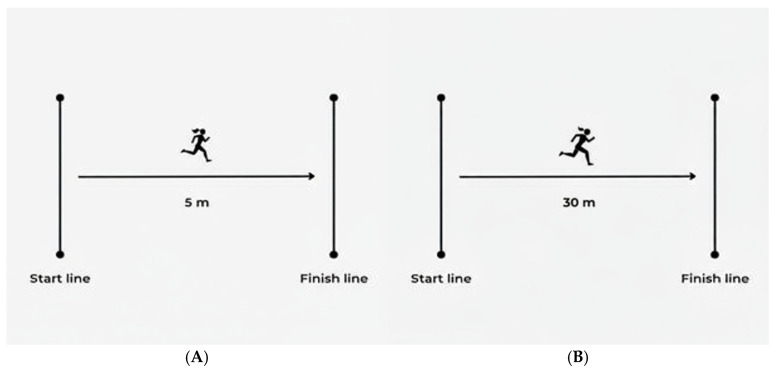
Schematic presentation of the 5 m (**A**) and 30 m (**B**) sprint test. Circles represent the position of photocells.

Running times were recorded using two pairs of dual-beam Witty Gate photocells (Microgate, Bolzano, Italy), a valid and reliable system for sprint performance assessment [24]. Following a standardized warm-up, participants performed two maximal 30-m sprint trials on an indoor artificial grass surface, with 15 min of passive recovery between trials. To prevent premature activation of the timing system, participants started with the leading foot positioned 0.5 m behind the first timing gate. Split times at 5 m and 30 m were recorded automatically, and the best performance from the two trials was retained for statistical analysis (Figure 2).

### 2.7. RAST (Running-Based Anaerobic Sprint Test)

**Figure 3:**
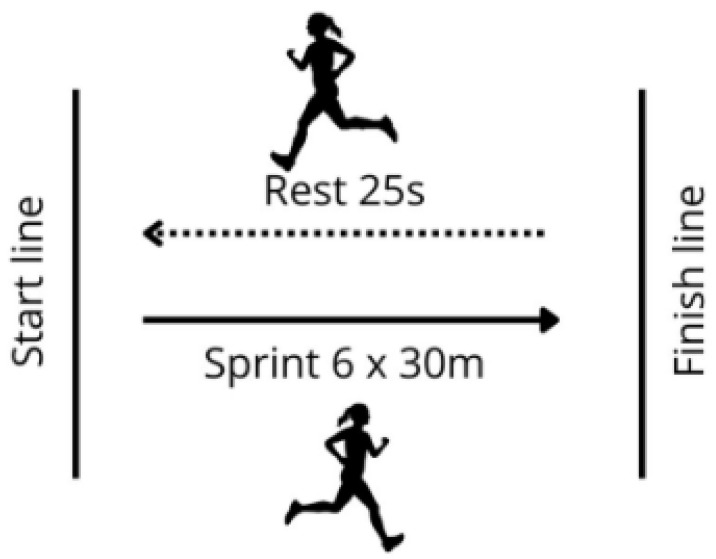
Schematic presentation of the RAST.

The RAST protocol involved six maximal 30-m sprint efforts separated by 25 s of active recovery [25]. Sprint times were recorded using infrared photocell gates (Witty, Microgate System, Mahopac, NY, USA) positioned at the start and finish lines of each sprint. The tests were performed on a 30-m indoor athletics track under standardized environmental conditions. The total time required to complete the six 30-m sprints (Σ6 × 30 m) was used as an indicator of repeated sprint ability and anaerobic performance and was retained for statistical analysis. Before testing, the participants completed a standardized 15-min warm-up consisting of light jogging, dynamic stretching, and progressive accelerations. Following a 5-min passive recovery, the athletes performed the RAST protocol. They were instructed to sprint each 30-m distance at maximal effort, decelerate beyond the finish line, and return to the starting line during the recovery interval before commencing the next repetition. The procedure was repeated until all six sprints had been completed (Figure 3).

### 2.8. Countermovement-Jump (CMJ) Assessment

Countermovement jump performance was assessed using dual force plates (ForceDecks, VALD Performance, Brisbane, Australia), a validated system for quantifying vertical jump kinetics and kinematics. The testing protocol was adapted from the methodology described by Gepfert et al. [26]. Before each trial, participants stood quietly on the force plates for 3 s to determine body mass and baseline force. Testing consisted of bilateral countermovement jumps with arm swing (CMJa) followed by unilateral countermovement jumps (single-leg CMJ) performed separately for the left and right limbs. During the bilateral CMJa, participants stood with their feet shoulder-width apart and performed a rapid downward movement to a self-selected countermovement depth before immediately jumping vertically with maximal effort while using an unrestricted arm swing. Three maximal trials were completed, with 1 min of passive recovery between attempts. The trial with the highest peak power output was retained for further analysis.

During the single-leg CMJ, participants performed the test separately on the dominant and non-dominant limbs. The starting position consisted of standing on one leg with the trunk upright and the free limb flexed at approximately 90° at the hip and knee. Participants performed a rapid countermovement followed immediately by a maximal vertical jump while keeping the testing leg on the force plate throughout the movement. Three maximal trials were performed for each limb with 30 s of passive recovery between attempts. The trial with the greatest jump height for each limb was retained for statistical analysis. Vertical ground reaction force data were sampled at 1000 Hz and analyzed using the manufacturer’s software. The following variables were extracted: bilateral jump height (cm), peak power (W·kg^−1^), concentric peak force (N·kg^−1^), unilateral jump height (cm), and unilateral peak power (W).

### 2.9. Statistical Analysis

The required sample size was estimated a priori using G*Power 3.1.9.7 [27] for the *F*-test family, repeated-measures ANOVA testing the within–between (group × time) interaction of a 2 (group) × 2 (pre/post) mixed design. With *α* = 0.05, power (1 − *β*) = 0.80, effect size *f* = 0.30, two groups, two measurements, correlation among repeated measures *r* = 0.65, and *ε* = 1, the analysis yielded a minimum total sample size of *N* = 18 (*λ* = 9.26; critical *F* = 4.49; df = 1, 16; actual power = 0.81). Accordingly, 18 female professional soccer players were enrolled in this study and randomly allocated to two equal groups (*n* = 9 each). A power of 0.80 (*β* = 0.20) at *α* = 0.05 follows the conventional 4:1 balance between type II and type I errors [28], a standard that remains feasible in elite athlete research, where the accessible population is inherently small (median sample in sports science ≈ 19) [29]. The effect size *f* = 0.30 represents a moderate-to-large effect on Cohen’s scale (0.10/0.25/0.40 = small/medium/large) [28], chosen as the smallest practically and physiologically meaningful group × time interaction expected from a nutritional intervention in already highly trained athletes. The correlation among repeated measures (*r* = 0.65) was set conservatively, consistent with the moderate-to-good test–retest reliability of performance and physiological measures typically reported in athletes (ICC ≥ 0.63) [30,31]; with only two measurement occasions, sphericity was held, and no correction was applied (*ε* = 1). All analyses were performed using the Statistica 13.1 package. The normality of the distributions was verified using the Shapiro–Wilk test, Levene’s test was used to verify the homogeneity of variances, and the Mauchley test was used to verify sphericity. The results are presented as means with standard deviations, standard errors, and 95% confidence intervals. A multi-criterial repeated measures ANOVA was used to compare the differences between the considered variables. The effect sizes for the main effects and interactions were determined using partial eta squared (η^2^). The ESs were classified as small (0.01–0.059), moderate (0.06–0.137), and large (>0.137). In the case of significant differences for the main effect or interaction, post hoc comparisons were conducted using Bonferroni’s post hoc test. The statistical significance for the differences between the type of load and muscle side was set at *p* < 0.05. Effect sizes (Cohen’s d) were also calculated. The ES was interpreted as large for d > 0.8, moderate for d between 0.8 and 0.5, and small for d < 0.5. To analyze the significance of differences between effects, depending on the normality of distributions, the *t*-test for independent samples or the Mann–Whitney U test was used. Justification of the assumed effect size: the a priori *f* = 0.30 was selected as the smallest group × time interaction judged practically and physiologically meaningful for a nutritional intervention in already highly trained athletes, rather than as the effect anticipated from previous supplementation trials. Because several vitamin D supplementation studies have reported smaller performance effects, this assumption may be optimistic; the analysis was therefore powered to detect moderate-to-large, but not small, intervention effects, and this limitation is acknowledged in the Discussion. Because multiple outcome variables were analysed, no formal correction for multiple comparisons (e.g., false discovery rate) was applied across the full family of outcomes; Bonferroni correction was used only for post hoc pairwise comparisons following a significant ANOVA effect. Isolated significant findings should therefore be interpreted with caution, and the increased risk of type I error is acknowledged.

## 3. Results

Results are reported as statistical outcomes; physiological and practical interpretation is reserved for the Discussion.

Table 2 presents descriptive statistics for vitamin D metabolites and hematological variables. At baseline, after summer exposure, four players (22.2%) had total serum 25(OH)D concentrations ≤ 30 ng/mL, whereas 14 players (77.8%) had concentrations > 30 to 50 ng/mL; none exceeded 50 ng/mL.

Change in vitamin D status. The mean within-group change (Δ) in total serum 25(OH)D was +0.8 ng/mL in the supplementation group (36.7 to 37.4 ng/mL) and +4.9 ng/mL in the placebo group (37.6 to 42.5 ng/mL). The corresponding changes in 1,25(OH)_2_D were +0.6 pg/mL and −9.1 pg/mL, respectively. The group × time interaction for total serum 25(OH)D was not statistically significant (F = 0.52; *p* = 0.47; η*p*^2^ = 0.030).

No significant group × time interactions were observed for total serum 25(OH)D (F = 0.52; *p* = 0.47; η*p*^2^ = 0.030), 1,25(OH)_2_D (F = 0.74; *p* = 0.40; η*p*^2^ = 0.042), RBC (F = 0.47; *p* = 0.50; η*p*^2^ = 0.026), Hb (F = 3.04; *p* = 0.09; η*p*^2^ = 0.15), WBC (F = 0.26; *p* = 0.87; η*p*^2^ = 0.001), lymphocytes (F = 1.51; *p* = 0.24; η*p*^2^ = 0.08), basophils (F = 2.59; *p* = 0.13; η*p*^2^ = 0.13), neutrophils (F = 1.20; *p* = 0.28; η*p*^2^ = 0.066), or eosinophils (F = 0.38; *p* = 0.54; η*p*^2^ = 0.02). Significant main effects of time were observed for Hb (*p* < 0.001) and lymphocyte count (F = 7.99; *p* = 0.011; η*p*^2^ = 0.082); the remaining time effects were not statistically significant.

### Performance Outcomes

Table 3 presents descriptive statistics and standardized within-group effect sizes for the performance outcomes. No significant group × time interactions were observed for the 5-m sprint, 30-m sprint, RAST total sprint time, bilateral CMJ peak power, concentric peak force, bilateral jump height, unilateral peak power, or right-leg jump height (all *p* > 0.05). Significant main effects of time were observed for 30-m sprint performance (F = 10.26; *p* = 0.005; η*p*^2^ = 0.38), RAST total sprint time (F = 16.55; *p* = 0.001; η*p*^2^ = 0.49), bilateral jump height (F = 9.65; *p* = 0.006; η*p*^2^ = 0.36), left-leg peak power (F = 154.01; *p* < 0.001; η*p*^2^ = 0.90), right-leg jump height (F = 14.8; *p* = 0.001; η*p*^2^ = 0.46), and left-leg jump height (F = 9.18; *p* = 0.007; η*p*^2^ = 0.35). Significant main effects of group were observed for RAST total sprint time (F = 4.53; *p* = 0.04; η*p*^2^ = 0.21) and bilateral jump height (F = 5.38; *p* = 0.033; η*p*^2^ = 0.24). A significant group × time interaction was observed only for left-leg jump height (F = 6.16; *p* = 0.024; η*p*^2^ = 0.26). Given the number of outcomes analysed, this isolated interaction was treated as exploratory and was not interpreted as evidence of a supplementation-specific effect.

## 4. Discussion

The present study assessed total serum 25(OH)D concentrations in professional female soccer players and examined whether eight weeks of vitamin D_3_ supplementation at 4000 IU/day produced additional changes in vitamin D status, hematological variables, sprint and repeated-sprint performance, and countermovement-jump outcomes. The principal finding was that supplementation was not associated with additional performance improvements compared with placebo under the conditions examined.

### 4.1. Vitamin D Concentrations

At baseline, 25% of the participants had serum 25(OH)D concentrations of ≤30 ng/mL. This finding suggests that even during summer at approximately 50° N, sunlight exposure may not be sufficient to maintain adequate vitamin D status in all athletes. Our findings are broadly consistent with Braun et al. [32], who reported insufficient 25(OH)D concentrations in 38% of 56 young German female soccer players. In our earlier study of male professional soccer players assessed after summer, 7% had deficient concentrations, 43% had insufficient concentrations, and 50% had concentrations between 30 and 50 ng/mL [14]. Together with reports from other soccer populations, these observations indicate that insufficient vitamin D status may persist in athletes even after periods of outdoor summer training [12,13,14,32]. Although neither group showed a statistically significant change in 25(OH)D, the study was likely underpowered to detect between-group differences in vitamin D status of the magnitude observed; the small sample (*n* = 9 per group) limited sensitivity to moderate intervention effects, and a non-significant result should not be interpreted as evidence of no effect. The observed changes in 25(OH)D did not differ significantly between groups, and the magnitude of change was small in the supplementation group; nevertheless, the study had limited statistical power to detect moderate between-group differences in vitamin D status. The present results should therefore be regarded as hypothesis-generating, and adequately powered trials are required to determine whether 4000 IU/day meaningfully alters 25(OH)D status in athletes with predominantly sufficient baseline concentrations.

### 4.2. Vitamin D and Hematological Parameters

In addition to evaluating serum 25(OH)D concentrations, we examined selected hematological variables. Vitamin D_3_ supplementation at 4000 IU/day was not associated with differential changes in erythrocyte count, hemoglobin concentration, or the selected white blood cell subtypes. Although vitamin D has been implicated in erythropoiesis and immune regulation, the absence of measurable between-group effects after eight weeks may reflect the relatively short intervention, the predominantly sufficient baseline vitamin D status, and the influence of other physiological determinants of hematopoiesis. These findings should be interpreted cautiously because ferritin, iron status, vitamin B_12_, folate, hydration status, menstrual blood loss, and inflammatory markers were not assessed. Longer interventions incorporating a more comprehensive evaluation of hematological determinants are therefore warranted [8].

### 4.3. Vitamin D and Performance

The primary performance finding was that vitamin D_3_ supplementation at 4000 IU/day for eight weeks was not associated with additional improvements in 5-m or 30-m sprint performance, RAST total sprint time, or countermovement-jump outcomes compared with placebo. These findings are limited to the outcomes assessed, because soccer performance is multidimensional and depends on reaction time, perceptual–motor efficiency, agility, and decision-making. Mancini et al. [17] reported associations between visual reaction time and field-based agility in young athletes, supporting a broader interpretation of soccer performance than linear sprint and jump measures alone.

No clear differences in training-related adaptations were observed between players with baseline 25(OH)D concentrations ≤30 ng/mL and those with concentrations >30 ng/mL. Improvements observed over time in sprint, repeated-sprint, and jump outcomes were therefore more consistent with adaptations to the common training programme than with a supplementation-specific effect. However, the small number of participants with insufficient vitamin D status limited the ability to determine whether baseline status modified the response to supplementation. Direct comparison with previous research is difficult because randomized controlled trials of vitamin D supplementation in professional female soccer players are lacking. Observational studies in female players have produced mixed findings. Brännström et al. [2] found no consistent associations between 25(OH)D concentrations and most muscle performance outcomes, although higher concentrations were associated with a shorter time to peak knee extension power. Lozano-Berges et al. [3] reported that players with sufficient baseline vitamin D status showed more favorable changes in selected bone and strength outcomes over a season, whereas players with insufficient status experienced declines in some performance measures. These observational associations do not demonstrate that supplementation itself improves performance.

A biological relationship remains plausible because vitamin D signaling may influence calcium handling, muscle protein turnover, and mitochondrial metabolism [7,33,34,35,36,37,38,39]. Nevertheless, these mechanisms were not directly assessed in the present study and should not be regarded as confirmed explanations for the observed outcomes. The absence of additional performance benefits suggests that increasing serum 25(OH)D concentrations in athletes with generally sufficient baseline status may not necessarily translate into measurable improvements in sprint or power performance.

The present findings should also be considered alongside our previous randomized trial in professional male soccer players, in which supplementation with 6000 IU/day was associated with more favorable changes in vitamin D status and selected performance outcomes [14]. Although differences in sex, baseline vitamin D status, seasonal timing and sunlight exposure, supplementation dose and duration, training load, and overall study design preclude direct comparison between the two studies, the findings suggest that the response to vitamin D supplementation may depend on both baseline vitamin D status and the supplementation regimen. According to the UEFA expert group statement, vitamin D_3_ supplementation at 2000 IU/day may be considered when deficiency is identified, followed by reassessment of serum 25(OH)D concentrations to evaluate the individual response [40]. Although concentrations above 30 ng/mL are generally considered sufficient, a target of approximately 40 ng/mL has been proposed in parts of the sports nutrition literature [38,41,42]. This threshold is not universally accepted, and evidence that concentrations above 40 ng/mL provide additional performance benefits remains insufficient. Supplementation should therefore be individualized according to baseline status and accompanied by biochemical monitoring. Further adequately powered randomized trials are required to determine the optimal dose and duration and to establish whether correcting vitamin D deficiency improves neuromuscular performance in female athletes.

### 4.4. Strengths and Limitations

The present study had several important strengths. The randomized, double-blind, placebo-controlled design reduced the risk of bias and strengthened internal validity. The investigation included a well-defined and underrepresented population of professional female soccer players with comparable training backgrounds and competitive demands. All participants followed the same team training programme, reducing variability in training exposure. The standardized supplementation protocol (4000 IU/day) and the use of validated performance assessments, including countermovement jumps, 5-m and 30-m sprint tests, and a repeated-sprint protocol, further strengthened the study design. Moreover, assessment of total serum 25(OH)D and 1,25(OH)_2_D concentrations enabled biochemical evaluation of the response to supplementation. Importantly, the findings indicate that, under the conditions examined, vitamin D_3_ supplementation was not associated with additional improvements in physical performance compared with placebo.

Nevertheless, several limitations of this study should be acknowledged. The relatively small sample size limited the statistical power to detect small or moderate intervention effects, while the eight-week intervention period may have been insufficient to produce measurable changes in the selected performance and hematological outcomes. The trial was retrospectively registered. Supplementation adherence was regularly monitored by club staff; however, it was not objectively quantified using capsule counts, supplementation diaries, or electronic monitoring. Because the participants were drawn from an elite professional population, the accessible sample was necessarily small, and the study had limited statistical power to detect small-to-moderate intervention effects; the absence of significant supplementation effects should be interpreted in this context rather than as definitive evidence of no effect.

Dietary vitamin D and calcium intakes and individual sunlight exposure were not systematically assessed. Data on skin phototype, sunscreen use, travel history, and ultraviolet exposure before enrolment were also unavailable. The mixed baseline vitamin D status, with a substantial proportion of participants already presenting sufficient total serum 25(OH)D concentrations, may have reduced the potential for observing additional performance-related benefits in this study. Furthermore, only total serum 25(OH)D was measured, whereas free and bioavailable 25(OH)D and vitamin D-binding protein was not assessed.

The menstrual cycle phase was not controlled during performance assessments, and information concerning hormonal contraceptive use, menstrual dysfunction, and menstrual blood loss was not systematically collected. Body composition was assessed using bioelectrical impedance analysis rather than a reference method, such as DXA, and hydration status was not objectively verified. The interpretation of the hematological findings was additionally limited by the absence of ferritin, iron status, vitamin B12, folate, parathyroid hormone, calcium, magnesium, inflammatory markers, and indicators of muscle damage. Finally, the number of statistical comparisons may have increased the risk of type I error, and recruitment from a single professional team limits the generalizability of the findings.

Future studies should include larger cohorts, preferably female athletes with confirmed vitamin D insufficiency or deficiency, longer intervention periods, objective assessment of adherence, dietary intake, sunlight exposure, and more comprehensive biochemical and menstrual status monitoring.

### 4.5. Generalizability of the Findings

The findings of this randomized controlled trial should be interpreted within the context of the studied population, which consisted of professional female soccer players recruited from a single team. Therefore, the results are primarily applicable to elite female athletes exposed to similar training loads, competitive demands, seasonal conditions, and baseline vitamin D status. Eight weeks of vitamin D_3_ supplementation at a dose of 4000 IU/day was not associated with additional improvements in speed or power performance compared with placebo under the conditions examined.

These findings should not be directly extrapolated to male athletes, younger or older populations, recreational players, athletes from other sports disciplines, or individuals with a confirmed vitamin D deficiency. Sex-specific physiological characteristics, hormonal status, training level, baseline 25(OH)D concentration, and neuromuscular responses may influence the effects of the supplementation. The applicability of the findings is also limited to a similar intervention duration and late-summer/early autumn conditions, during which endogenous vitamin D synthesis may differ from that in winter. Further studies involving larger and more diverse athletic populations, particularly female athletes with confirmed vitamin D insufficiency or deficiency, and longer intervention periods are required to confirm these findings.

## 5. Conclusions

Eight weeks of vitamin D_3_ supplementation at 4000 IU/day was not associated with additional improvements in muscular strength, sprint performance, repeated-sprint ability, or countermovement-jump outcomes compared with placebo in professional female soccer players. The improvements observed over time were more consistent with adaptations to the common training programme than with a supplementation-specific effect. Approximately 25% of participants had insufficient vitamin D status after the summer period, indicating that suboptimal 25(OH)D concentrations may occur in elite female soccer players despite seasonal sunlight exposure. These findings support regular monitoring of vitamin D status and individualized supplementation when insufficiency or deficiency is identified, rather than routine escalation of the dose in all athletes. Interpretation should consider the small sample size and the generally sufficient baseline vitamin D status of most participants. Larger randomized controlled trials involving female athletes with confirmed vitamin D insufficiency or deficiency, longer intervention periods, and objective monitoring of adherence, dietary intake, sunlight exposure, and individual biochemical responses are needed to clarify whether supplementation provides additional performance benefits.

## Figures and Tables

**Table 1:** Training scheme during the 8-week study.

Numbers of Microcycles	Days of the Week						
	Monday	Tuesday	Wednesday	Thursday	Friday	Saturday	Sunday
1	F	G + F	F	G + F	F	S	DO
2	F	G + F	S	G + F	F	S	DO
3	F	G + F	S	G + F	F	S	DO
4	F	G + F	F	G + F	F	S	DO
5	F	G + F	F	F	F	LG	DO
6	F	G + F	F	F	F	LG	DO
7	F	G + F	F	F	F	LG	DO
8	F	G + F	F	F	F	LG	DO

Note: F, training in the field; G + F, gym + training in the field; S, sparring; LG, league game; DO, day off.

**Table 2:** Changes in vitamin D metabolites and hematological parameters in the supplementation and placebo groups.

Variables	SG		PG	
	Before	After	Before	After
	M ± SD 95% CI	M ± SD 95% CI	M ± SD 95% CI	M ± SD 95% CI
Total 25(OH)D [ng/mL]	37.60 ± 8.09 31.82; 43.38	42.50 ± 8.97 36.08; 48.92	36.67 ± 7.68 30.76; 42.57	37.44 ± 9.62 30.05; 44.84
1,25(OH)_2_D [pg/mL]	60.61 ± 13.44 50.99; 70.23	51.50 ± 14.35 41.23; 61.77	53.79 ± 11.71 44.79; 62.79	54.34 ± 15.58 42.37; 66.32
RBC [×10^6^/µL]	4.39 ± 0.32 4.17; 4.62	4.50 ± 0.23 4.33; 4.66	4.37 ± 0.18 4.23; 4.51	4.35 ± 0.17 4.22; 4.48
Hb [g/dL]	13.42 ± 0.67 12.94; 13.90	13.64 ± 0.61 13.20; 14.08	12.87 ± 0.41 12.55; 13.18	13.64 ± 0.48 13.27; 14.02
WBC [×10^3^/µL]	5.73 ± 0.93 5.07; 6.40	6.30 ± 1.37 5.32; 7.28	6.12 ± 1.36 5.07; 7.16	6.54 ± 1.30 5.54; 7.54
Lymphocytes [×10^3^/µL]	2.45 ± 0.46 2.13; 2.78	2.77 ± 0.58 2.35; 3.18	2.07 ± 0.42 1.75; 2.39	2.86 ± 0.78 2.26; 3.46
Neutrophils [×10^3^/µL]	2.43 ± 0.51 2.06; 2.79	2.75 ± 0.97 2.05; 3.44	3.25 ± 1.19 2.33; 4.17	2.88 ± 0.63 2.40; 3.37
Basophils [×10^3^/µL]	0.044 ± 0.019 0.030; 0.058	0.034 ± 0.012 0.026; 0.042	0.032 ± 0.017 0.019; 0.045	0.042 ± 0.024 0.023; 0.061
Eosinophils [×10^3^/µL]	0.16 ± 0.082 0.092; 0.22	0.17 ± 0.061 0.13; 0.22	0.22 ± 0.14 0.12; 0.32	0.19 ± 0.12 0.10; 0.27

Note: M, mean; SD, standard deviation; CI, confidence interval; RBC, red blood cell count; Hb, hemoglobin; WBC, white blood cell count; SG, supplementation group; PG, placebo group. Individual participant-level 25(OH)D values are available from the corresponding author.

**Table 3:** Changes in performance outcomes in the supplementation and placebo groups.

Outcome			SG		PG		Cohen’s d ^†^
			Before	After	Before	After	SG; PG
			M ± SD 95% CI	M ± SD 95% CI	M ± SD 95% CI	M ± SD 95% CI	(Pre–Post)
5-m sprint [s]			1.23 ± 0.071 1.18; 1.28	1.20 ± 0.061 1.15; 1.24	1.24 ± 0.064 1.19; 1.29	1.23 ± 0.078 1.17; 1.29	−0.45; −0.14
30-m sprint [s]			4.72 ± 0.16 4.60; 4.84	4.61 ± 0.14 4.50; 4.71	4.82 ± 0.19 4.67; 4.96	4.74 ± 0.17 4.62; 4.87	−0.73; −0.44
RAST total sprint time (Σ6 × 30 m) [s]			29.26 ± 1.09 28.48; 30.04	28.60 ± 0.69 28.11; 29.09	30.18 ± 1.12 29.31; 31.04	29.50 ± 1.03 28.70; 30.29	−0.72; −0.63
Bilateral CMJ peak power [W/kg]			62.65 ± 11.85 54.17; 71.13	62.42 ± 12.01 53.83; 71.01	49.03 ± 6.82 43.79; 54.28	56.00 ± 15.14 44.37; 67.63	−0.02; +0.59
		Bilateral CMJ concentric peak force [N/kg]	25.41 ± 2.42 23.68; 27.14	27.11 ± 2.18 25.55; 28.67	24.82 ± 2.68 22.76; 26.88	24.72 ± 2.29 22.96; 26.48	+0.74; −0.04
		Bilateral CMJ jump height [cm]	36.14 ± 3.85 33.39; 38.89	38.80 ± 4.33 35.70; 41.90	32.67 ± 3.51 29.97; 35.36	35.39 ± 3.05 33.04; 37.73	+0.65; +0.83
	Single-leg CMJ left-leg jump height [cm]		22.47 ± 2.80 20.47; 24.47	22.61 ± 2.54 20.79; 24.43	19.94 ± 3.68 17.12; 22.77	21.36 ± 3.28 18.83; 23.88	+0.05; +0.41
		Single-leg CMJ right-leg jump height [cm]	21.17 ± 4.45 17.98; 24.36	22.42 ± 3.79 19.71; 25.13	20.37 ± 3.23 17.89; 22.85	21.51 ± 2.49 19.60; 23.43	+0.30; +0.40
		Single-leg CMJ left-leg peak power [W]	2138.60 ± 741.77 1607.97; 2669.23	2168.10 ± 760.24 1624.26; 2711.94	2110.00 ± 557.10 1681.77; 2538.23	2356.11 ± 650.18 1856.34; 2855.88	+0.04; +0.41
		Single-leg CMJ right-leg peak power [W]	2094.40 ± 760.46 1550.40; 2638.40	2086.20 ± 741.57 1555.71; 2616.69	2205.22 ± 413.03 1887.74; 2522.71	2641.78 ± 708.22 2097.39; 3186.16	−0.01; +0.75

Note: RAST, Running-Based Anaerobic Sprint Test; SG, supplementation group; PG, placebo group. ^†^ Cohen’s d for the within-group pre–post-change, calculated as the mean change divided by the pooled pre- and post-intervention standard deviation. Absolute d values of approximately 0.2, 0.5, and 0.8 indicate small, moderate, and large effects, respectively. These within-group effect sizes do not represent the between-group treatment effect.

## Data Availability

The original contributions presented in this study are included in the article. Further inquiries can be directed to the corresponding author.

## Supplementary Materials

The following supporting information can be downloaded at: https://www.mdpi.com/article/10.3390/nu18152460/s1, Figure S1: CONSORT 2010 flow diagram of participant enrollment, allocation, follow-up, and analysis.
