# Supplementary material for: Eight-Week Vitamin D3 Supplementation at 4000 IU/Day Was Not Associated with Further Improvements in Speed or Power Performance in Professional Female Soccer Players: A Randomized Controlled Trial
Source: Nutrients. 2026 Jul 28;18(15):2460. doi: 10.3390/nu18152460 (PMC13467743; doi:10.3390/nu18152460)

Figure S1. CONSORT 2010 flow diagram of participant enrollment, allocation, follow-up, and analysis.

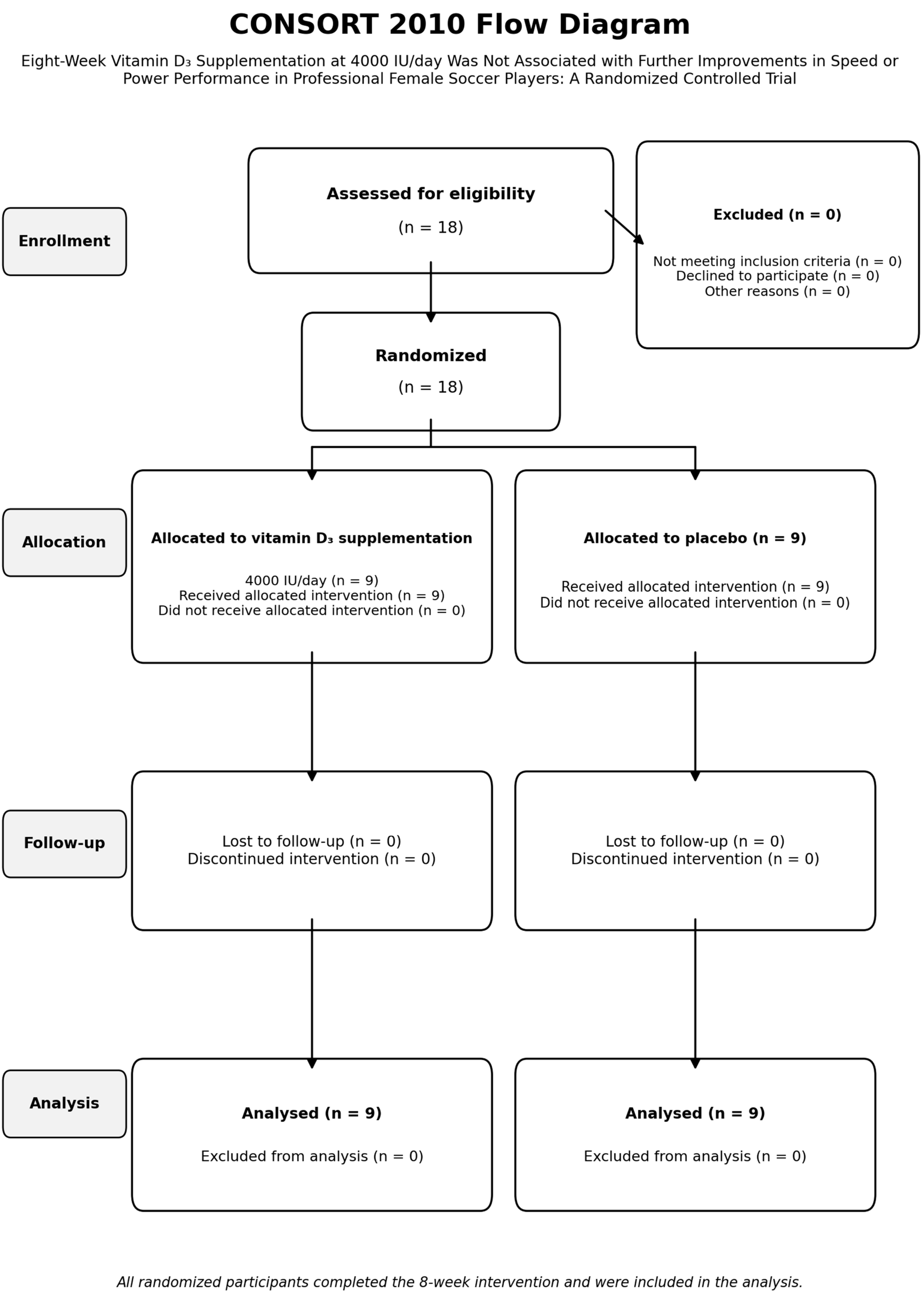

Supplement: Supplementary file 1 [file nutrients-18-02460-s001.zip › nutrients-4426904-supplementary.pdf]
